# Newborn Health Indicators Associated with Maternal Age during First Pregnancy

**DOI:** 10.3390/ijerph16183448

**Published:** 2019-09-17

**Authors:** Leticia Molina-García, Manuel Hidalgo-Ruiz, Ana María Cámara-Jurado, Maria Jose Fernández-Valero, Miguel Delgado-Rodríguez, Juan Miguel Martínez-Galiano

**Affiliations:** 1 Complejo Hospitalario de Jaén, 23007 Jaén, Spain; letitedi@gmail.com (L.M.-G.); mariajosefernandezvalero@gmail.com (M.J.F.-V.); 2 Hospital San Juan de la Cruz, 23400 Úbeda, Spain; manuel9282@gmail.com; 3 Hospital San Agustín, 23700 Linares, Spain; ancaju144@yahoo.es; 4 Department of Health Sciences, University of Jaen, 23071 Jaen, Spain; mdelgado@ujaen.es; 5 Consortium for Biomedical Research in Epidemiology and Public Health (CIBERESP), 28029 Madrid, Spain; 6 Department of Nursing, Campus de Las Lagunillas s/n, Edificio B3, University of Jaen, 23071 Jaen, Spain

**Keywords:** maternal age, neonatal outcomes, child, breastfeeding

## Abstract

Delaying motherhood is becoming increasingly common, raising questions of the possible influence that maternal age may have on newborn health. Therefore, the objective of this study was to determine the association between maternal age and different newborn health parameters. An observational study was conducted in Spain on primiparous women and their infants. Data were collected on newborn health variables, breastfeeding, and different clinical practices that are beneficial for child health and development. Crude and adjusted mean differences were calculated along with the standard error of the mean. A total of 373 women and their children participated. In terms of early commencement skin-to-skin contact, the mean age of women that did skin-to-skin contact was 29.95 ± 0.31 years compared to 31.49 ± 0.66 years in those that did not (*p* = 0.042). In terms of other newborn parameters, such as preterm birth, health problems or complications, or the need for hospital admission, these were more frequent in the oldest group of mothers, but the differences found were not significant (*p* > 0.05). Hence, indicators of newborn morbidity were not found to be significantly associated with maternal age; however, beneficial practices such as early commencement skin-to-skin contact were found to be significantly associated with maternal age.

## 1. Introduction

The age at which a woman will have her first children is increasing [1,2]; this delay in motherhood has been studied in terms of obstetric outcomes, such as the development of any pathologies during pregnancy, the type of birth, as well as other parameters related to the pregnancy, labor, and delivery [3,4]. However, the impact of maternal age on newborn’s health has not been as well studied, with most research focusing on the effect of advanced maternal age (mother’s age over 35 years, 40 years or more) but not including any age [5,6,7,8,9,10,11,12,13,14,15,16]. A case–control study conducted in Sweden on 109,030 women who gave birth between 2007 and 2013 concluded that an advanced maternal age was associated with a greater probability of a premature newborn, low birth weight, low Apgar scores at 5 and 10 min, and higher mortality during the first month of life [5]. In a retrospective cohort study conducted in Thailand with 19,599 women, an advanced maternal age was associated with adverse perinatal outcomes such as a preterm birth and intrauterine growth restriction [6].

In recent years, the benefits of delayed motherhood have also been studied [12,13,14,15,16]. In Argentina, a descriptive retrospective cross-sectional study conducted with 170 women and infants aged between 6 and 12 months found that maternal age influenced the duration of breastfeeding; only 25% of mothers younger than 20 years maintained exclusive breastfeeding until 6 months, which increased to 75% in older mothers [12]. In the United Kingdom, a 2017 study using data from three British studies concluded that infants born when mothers were older than 35 years showed better cognitive skills than those of younger mothers [13].

However, the studies conducted to date do not show consistent results for infant health parameters and beneficial health practices such as the early initiation of breastfeeding and early skin-to-skin contact [5,6,7,8,9,10,11,12,13,14,15,16,17]. We considered that the age at which a woman becomes a mother for the first time may influence the implementation of the different practices recognized as beneficial for the newborn and its health parameters. Therefore, the objective of this study was to establish the association between maternal age and different newborn health parameters, including nutritional data, clinical data on wellbeing, and aggregate outcome measures.

## 2. Materials and Methods

An observational analytical study was conducted on pregnant women and their babies that were delivered during 2017 in different hospitals in South Spain. Inclusion criteria were primiparous women with a singleton pregnancy and who were not minors (≥18 years). Women who had difficulty communicating in Spanish were excluded (language barrier).

Ethics committee approval for this study was obtained from all the participating hospitals in this study. Informed consent was obtained from the participating women and the established protocols of each medical center were followed for access to medical record data and to conduct this type of research.

The principal outcome was the appearance of pathology associated with the pregnancy. According to the 2011 study by Heras Pérez et al. [3], the incidence of pregnancy-associated pathology in women older than 35 years was 29.2% compared to 15.8% in the group of women 35 years or younger; for the detection of significance (*p* < 0.05) with a statistical power of 80%, a sample size of 302 women was necessary. Considering a drop-out rate of 15%, 373 women were recruited. Women were selected consecutively, with a sample size proportional to the specific weight of each center.

### 2.1. Data Collection

Data were collected using a previously piloted questionnaire that was administered by qualified and knowledgeable personnel via an interview two hours after childbirth and at hospital discharge, and via a telephone call two months postpartum. The majority of the data were obtained in the clinical interview and phone call conducted by health professionals following childbirth; however, the data were completed with access to the clinical history and the pregnancy and baby health documents.

Data were collected on the sociodemographic variables of the women (age, civil status, nationality, education level, income level, and employment during pregnancy), variables related to the newborn (gestational week of birth; skin-to-skin contact; anthropometric variables of weight, head circumference, and length; and Apgar scores), hospital admission (admission of the newborn immediately after birth in the neonatal unit or intensive care unit without staying with the mother for differing pathologies for which the pediatrician considers admission necessary, for example, respiratory distress, hypothermia, prematurity, etc.), health problems or perinatal complications (newborns with pathology that the pediatrician in the assessment of the newborn identifies as a health problem and collects it in the newborn’s medical history in the evaluation of the newborn that is done at different times either in the delivery room, maternity unit, or health centers, for example, tumors, respiratory distress, malformations, jaundice, cardiac arrhythmias, etc.) and the type of infant feeding.

### 2.2. Data Analysis

Continuous variables were assessed by comparison of means, *t*-test, or analysis of variance. The analysis of covariance was used to estimate adjusted means for potential confounders. For categorical variables, odds ratios (OR) and their 95% confidence intervals (CI) were computed using logistic regression to adjust for confounding. Confounders were considered those variables which were non-intermediate variables and changed the coefficient of the main exposure (maternal age) by more than 10% in multivariate models. Maternal pathology, maternal smoking habit, previous history of miscarriage, type of delivery and maternal sociodemographic variables were confounding variables in the multivariable analysis.

## 3. Results

A total of 373 women and their respective children participated. The mean age of the participating women was 30.45 ± 5.63 (18–47) years. In terms of civil status, 62.2% (n = 232) were married, and 27.3% (n = 102) were single. Almost all, 98.1% (n = 366), were Spanish. Eight women, 2.1%, had no education, 53.4% (n = 199) secondary education, and 37.8% (n = 141) had university level education. The mean income levels were 41.0% (n = 137) between 1000 and 1999 Euros per month, and 29.3% (n = 98) less than 1000 Euros per month. Around 71% (n = 265) worked during pregnancy, and 12.4% (n = 26) had some type of pathology prior to pregnancy. A previous history of miscarriage was present in 24.9% (n = 93). The pregnancy was planned in 87.4% (n = 325) of the women and required medical intervention to achieve pregnancy in 12.3% (n = 46). Antenatal education was taken by 58.4% (n = 218) of participants. Childbirth occurred at a mean gestation of 39.43 ± 1.41 weeks, as shown in Table 1, in which the characteristics of the study population are described.

In Table 2, we present the influence of maternal age on different infant parameters. The mean age of women that did skin-to-skin contact was 31.49 ± 0.66 years compared to 29.95 ± 0.31 years in those that did not (*p* = 0.042). In the results of the effect of maternal age on other beneficial practice, such as early initiation of breastfeeding, when adjusted for confusing variables (the education level of the mother, income level, smoking habit, previous history of miscarriage, type of delivery, and presence of any pathology prior to pregnancy), no significant difference was observed; the mothers who started breastfeeding early had a mean age of 30.12 ± 0.30 years compared to a mean age of 31.23 ± 0.73 years in mothers who did not start breastfeeding early (*p* = 0.164). In terms of other parameters related to the newborn and their hospital stay, such as health problems, complications, or the need for hospital admission, these were more frequent for newborn of older mothers; however, the differences found were not significant (*p* = 0.738, *p* = 0.174, *p* = 0.082, respectively). In the same way, health problems found in the newborn following hospital discharge and up to 2 months after were more frequent with older mothers (31.76 ± 1.08 years) than in younger mothers (30.16 ± 0.28 years), but the difference was also not significant (*p* = 0.154). No difference was found between maternal age and a small for gestational age infant (*p* = 0.866), low birth weight (<2500 g) (*p* = 0.488), and preterm birth (*p* = 0.324).

In Table 3, the associations between different variables related to the newborn, stratified by maternal age in four groups, can be seen: <25 years, 25–29 years, 30–34 years, and ≥35 years. Women who were ≥35 years had a negative association with performing skin-to-skin (ORa = 0.17; 95% CI: 0.04–0.71; *p* = 0.015).

In terms of skin-to-skin contact, the level of significance increased linearly with maternal age, with a significant association found in the age groups of 30–34 years and ≥35 years. The reference group was mothers younger than 25 years: 25–29 years (*p* = 0.053), 30–34 years (*p* = 0.041), and ≥35 years (*p* = 0.002). However, in terms of early commencement of breastfeeding, the association did not become significant for mothers ≥35 years (ORa = 0.55; 95% CI: 0.15–2.06; *p* = 0.374) compared to mothers younger than 25 years.

For the rest of the analyzed parameters, no association was observed between the different age groups.

Table 4 shows the associations between maternal age and different continuous newborn variables. The data did not show a significant difference for any of the parameters analyzed (*p* > 0.05), although we observed that mothers between 25 and 29 years were those that had newborns with higher anthropometric measures: weighed more (3310.56 ± 48.95 g), larger head circumference (34.71 ± 0.29 cm), longer (49.61 ± 0.32 cm), and had a higher Apgar score at 1 min (8.91 ± 0.06). The highest Apgar score at 5 min was observed in newborns of younger mothers (9.54 ± 0.06). Only 3 newborns had a score lower than 7 in the first minute of life, and none had a score lower than 7 at 5 min. No significant difference was found for any of these parameters.

## 4. Discussion

In our results, a younger maternal age is likely to favor skin-to-skin contact; a practice which has been demonstrated to be beneficial for infant health. Nevertheless, no difference was established between maternal age and other specific infant health parameters, such as low birth weight, small for gestational age, preterm birth, Apgar scores, hospital admission, complications following birth, anthropometric measures (weight, head circumference, and length), and the early initiation and maintenance of breastfeeding.

Early skin-to-skin contact, a recommended practice that provides multiple benefits [18], consists of placing the naked newborn in a ventral decubitus position on the mother’s bare chest just after birth or soon after. No studies were found that relate skin-to-skin contact with maternal age, therefore, we were unable to make any comparisons with our results. Nevertheless, there are two parameters (emotional bonding and attachment) that are closely related to early skin-to-skin contact; this was established by Guzmán Vela, who emphasized the influence of skin-to-skin contact in strengthening the emotional bond between the mother and infant [19], and also by Moore et al., who highlighted that skin-to-skin contact stimulates the secretion of hormones, such as oxytocin, that promote maternal bonding [20].

Mite Cárdenas and Pardo Torres, in a quantitative and descriptive study, compared the emotional bond between mothers and their newborns according to age and parity in 157 pregnancies distributed in three groups: adolescents (15–19 years), primiparous adults (≥20 years), multiparous adults (≥20 years). The comparison among the three groups showed that the participating adults (≥20 years) expressed a greater emotional bond with their infants compared to adolescent mothers (*p* = 0.02); significant differences were found for emotional support (*p* = 0.04) and union–interaction (*p* = 0.02) in favor of adult primiparous mothers and with disadvantage for adolescent mothers [21]. Giraldo Montoya et al., in a cross-sectional analytical study of 117 mothers and their children investigating demographic factors associated with attachment, found no association between maternal age and the pattern of attachment. Nonetheless, the adolescent mothers were found to have a higher percentage of insecure attachment compared to the rest—although this difference was not statistically significant (*p* = 0.255)—and the group of mothers aged 37 to 46 years presented a greater tendency to secure attachment [22].

Contrary to our results, Brasil Esteves et al. in their systematic review which included 18 studies on factors associated with breastfeeding in the first hour of life, identified a maternal age of less than 25 years as a risk factor, as well as lack of prenatal care, a low level of education of the mother, and cesarean delivery [17]. González et al., in a descriptive study using 170 surveys of mothers aged 14 to 45 years to assess the practice of exclusive breastfeeding, found that the age of the mother influenced the success or not of breastfeeding, with a lower rate of exclusive breastfeeding among adolescent mothers [12]. Estrada Rodríguez et al., in an analytic multicenter study conducted in Camagüey with 51 women in which a health education intervention was undertaken, also found a low rate of breastfeeding among adolescent mothers [23]. Oliver Roig et al. provided evidence that a maternal age older than 25 years is a protective factor for breastfeeding [24], and Schanler and Potak, in their systematic review, also found that an age younger than 25 years, and in particular, younger than 20 years, was a risk factor for not initiating breastfeeding [25].

In terms of the duration and maintenance of breastfeeding, González et al. concluded that the earlier breastfeeding was initiated, the longer exclusive breastfeeding was maintained, and that breastfeeding was extended to 6 months in 52% of the mothers that started during the first hour postpartum, decreasing to 33% in those that initiated breastfeeding in the first four hours postpartum, and only by 15% in those started after eight hours postpartum [12].

The benefit of the early onset of breastfeeding and early skin-to-skin contact has recently been included in the health policies of Spain as part of the humanization in childbirth care [26], however, this is not widely implemented in clinical practice. Access to this type of information via the internet may be easier and more common in younger mothers, and our results may reflect this.

Álvarez Caballero et al., in a descriptive cross-sectional study including 120 infants who were not exclusively breastfed until 6 months, identified factors that influenced the early abandonment of breastfeeding [27]. They highlighted as among the most frequent causes of early abandonment hypogalactia (49.1%), return to work or studies (24.1%), maternal illness (9.2%), breast refusal by the infant (9.2%), prolonged hospital admission for the infant (4.2%), infant was not gaining weight (2.5), and maternal weight loss (1.7%). These reasons were also documented in our results, however, no association with maternal age was observed.

Our results did not establish any significant difference between Apgar scores and maternal age. However, only three newborns had a score lower than seven in the first minute of life and none at five minutes. Moreover, the mean scores obtained in all age groups were higher than seven, indicating a good adaptation to extrauterine life following birth [28]. In contrast to our results, other authors have found a significant association between maternal age and Apgar scores [5,29,30,31]. Laffita Batista and Rodríguez Núñez et al. suggested that women older than 35 years have a higher number of pathologies that may alter the maternal–fetal gas exchange, which influences neonatal depression and, in turn, is reflected in the low Apgar scores [29,31]. A similar result was found by Machado and Hill, who also observed that a low Apgar score at birth occurred with a higher frequency in infants with mothers on both extremes of ages [30]. Sydsjö et al. also found lower Apgar scores in newborns of older mothers (40 years or older) [5].

Along the same lines as our results, Goisis et al., in his study of 124,098 children born during 1987–2000 in Finland, found that the association between an older maternal age and low birth weight or premature birth was not statistically significant [32]. In the same way, Kahveci et al., in a retrospective study conducted on 957 women, did not obtain significant differences for the rates of spontaneous premature births before 34 weeks and Apgar scores in the terms of maternal age [7]. However, they did conclude that late premature birth (34–37 weeks), low birth weight, and neonatal intensive care admission increased linearly with maternal age [7]. Wu et al. also associated maternal age with preterm birth in a study conducted in Canada [33]; the opposite to our results.

Sydsjö et al., in a study of 37,558 women who were 40 years or older that had given birth in Sweden during 2007–2012, found that the health of the newborn was often affected negatively in older mothers, with increased frequency of low birth weight, being small for gestational age, and health problems in the newborn during the first week [5]. In the same way, Ratnasiri Anura et al., in a retrospective cohort study of 5,267,519 births in California during the period 2005–2014, concluded that maternal age was a significant risk factor for low birth weight, independent of race or maternal ethnicity or education level [34]. Along the same lines and contrary to our results, Hidalgo-Lopezosa et al. associated maternal age with low birth weight in a study carried out in Spain [35], and Kahveci et al. found an association between a maternal age over 35 years and the incidence of SGA newborns [7].

Poor prenatal care has been associated with adverse neonatal outcomes such as low birth weight [36]. The women participating in this study mostly had adequate prenatal healthcare, with only 3 women having inadequate prenatal care. Prenatal care facilitates the early identification of any alteration of the normal course of pregnancy which may influence neonatal outcomes; this may have been one of the possible causes that an association between maternal age and neonatal outcomes has not been identified. Clinical surveillance with the objective of identifying the first signs of adverse outcomes may be a possible reason for our results.

As with all studies, we should comment on its strengths and limitations. The study sample was representative of the population. The questionnaire used to collect the data was previously piloted. The questions were clear and understandable for all levels of education, so an information bias can be ruled out. A selection bias associated with non-response is unlikely to have had an influence on our results, as the majority of the women selected participated, with only 13 who declined to participate and there were no indications that their response would have been different to those that agreed to participate. Finally, it is not possible to completely rule out the confounding bias inherent to observational studies. However, we believe that its effect on the study results was not significant as it was considered and controlled during the study design and development. This was done by using inclusion and exclusion criteria, as well as a multivariate analysis adjusted by confounding factors (found in the scientific literature and in clinical practice experiences) that could have influenced the results.

## 5. Conclusions

The age at which a woman has her first child influences the early commencement of skin-to-skin contact, with younger mothers carrying out these practices more frequently. No difference was found between a large number of newborn health problems or complications and maternal age, including Apgar scores, anthropometric measurements of the newborn, small for gestational age, low birth weight, or preterm birth. The mother’s age was not associated with the type of feeding (breastfeeding or artificial feeding) of the infant.

## Figures and Tables

**Table 1:** Study population characteristics (n = 373).

Variable	Value
Mean age (SD)	30.45 (5.63)
Civil status, n (%)	
Single	102 (27.3)
Married	232 (62.2)
De facto relationship	36 (9.7)
Divorced	3 (0.8)
Nationality, n (%)	
Spanish	366 (98.1)
Other	7 (1.9)
Education level, n (%)	
None	8 (2.1)
Primary	25 (6.7)
Secondary	105 (28.2)
Baccalaureate/Professional formation	94 (25.2)
University	141 (37.8)
Income level, n (%)	
<1000 Euros	98 (29.3)
1000–1999 Euros	137 (41.0)
2000–2999 Euros	68 (20.4)
≥3000 Euros	31 (9.3)
Employed during pregnancy, n (%)	
No	108 (29.0)
Yes	265 (71.0)
Presence of illness, n (%)	
No	326 (87.6)
Yes	46 (12.4)
Previous miscarriages, n (%)	
No	280 (75.1)
Yes	93 (24.9)
Planned pregnancy, n (%)	
No	47 (12.6)
Yes	326 (87.4)
Antenatal classes, n (%)	
No	155 (41.6)
Yes	218 (58.4)
Pregnancy care, n (%)	
Public health system	182 (48.8)
Private	7 (1.9)
Both	184 (49.3)
Medical assistance to achieve pregnancy, n (%)	
No	327 (87.7)
Yes	46 (12.3)
Gestational week of birth, mean (SD)	39.43 (1.41)
Infant weight (g), mean (SD)	3255.27 (434.21)
Newborn head circumference (cm), mean (SD)	30.46 (2.47)
Newborn length (cm), mean (SD)	49.46 (2.80)
Apgar score at 1 min, mean (SD)	8.84 (0.53)
Apgar score at 5 min, mean (SD)	9.44 (0.53)

**Table 2:** The influence of maternal age on different newborn health parameters.

Variable	Total, n	Crude Analysis		Multivariate Analysis *	
		Age of Mother m ± SEM	*p* Value	Age of Mother m ± SEM	*p* Value
Skin-to-skin			<0.001		0.042
No	75	32.48 ± 0.58		31.49 ± 0.66	
Yes	298	29.94 ± 0.33		29.95 ± 0.31	
Early initiation of breastfeeding			0.004		0.164
No	59	32.35 ± 0.76		31.23 ± 0.73	
Yes	305	30.08 ± 0.31		30.12 ± 0.30	
Small for gestational age			0.624		0.866
No	331	30.48 ± 0.31		30.25 ± 0.28	
Yes	40	30.02 ± 0.77		30.11 ± 0.82	
Low birth weight (<2500 g)			0.901		0.488
No	353	30.46 ± 0.30		30.31 ± 0.28	
Yes	20	30.30 ± 1.32		29.43 ± 1.23	
Preterm infant			0.253		0.324
No	361	30.39 ± 0.30		30.22 ± 0.27	
Yes	12	32.29 ± 1.76		31.78 ± 1.56	
Newborn health problems identified in the hospital			0.898		0.738
No	357	30.45 ± 0.29		30.24 ± 0.28	
Yes	16	30.63 ± 1.87		30.68 ± 1.27	
Complications in the newborn during the hospital stay			0.491		0.174
No	334	30.39 ± 0.31		30.14 ± 0.28	
Yes	39	31.04 ± 0.98		31.35 ± 0.84	
Hospital admission of the newborn			0.195		0.082
No	351	30.36 ± 0.30		30.14 ± 0.28	
Yes	22	31.97 ± 1.34		32.12 ± 1.10	
Exclusive breastfeeding during the first two months			0.441		0.831
No	196	30.24 ± 0.45		30.21 ± 0.38	
Yes	177	30.69 ± 0.36		30.33 ± 0.40	
Infant health problems during the first two months of life			0.064		0.154
No	350	30.32 ± 0.30		30.16 ± 0.28	
Yes	23	32.56 ± 1.39		31.76 ± 1.08	
Reason for abandonment of exclusive breastfeeding			0.557		
Did not abandon breastfeeding	187	30.75 ± 0.36		30.33 ± 0.39	ref.
Medical prescription	3	31.87 ± 1.80		30.96 ± 2.86	0.828
No milk	58	29.53 ± 0.88		30.58 ± 0.69	0.752
Infant remained hungry	85	30.96 ± 0.68		30.39 ± 0.57	0.926
Did not want to continue breastfeeding	8	28.94 ± 1.74		28.31 ± 1.89	0.297
Had to return to work	3	30.21 ± 1.47		28.57 ± 2.88	0.546
Other	29	29.22 ± 1.19		29.37± 1.00	0.378
Type of infant feeding during hospital stay			0.598		
Exclusively breastfed	214	30.47 ± 0.36		30.27 ± 0.36	ref.
Mixed	119	30.62 ± 0.57		30.10 ± 0.48	0.783
Formula	39	29.59 ± 0.88		30.41 ± 0.84	0.884

* Adjusted for the education level of the mother, income level, smoking habit, previous history of miscarriage, type of delivery, and presence of any pathology prior to pregnancy. Abbreviations: *m,* mean; SEM, standard error of mean.

**Table 3:** Association between stratified maternal age and newborn variables.

Variable	Age of Mother (Years)			
	<25 n (%)	25–29 n (%)	30–34 n (%)	≥35 n (%)
Skin-to-skin No Yes	3 (5.88) 48 (94.12)	16 (18.18) 72 (81.82)	25 (18.66) 109 (81.34)	31 (31.00) 69 (69.00)
OR, 95%CI *p* value	1 ref.	0.28 (0.08–1.02) 0.053	0.27 (0.08–0.95) 0.041	0.14 (0.04–0.48) 0.002
ORa *, 95%CI *p* value	1 ref.	0.27 (0.07–1.12) 0.072	0.29 (0.07–1.20) 0.088	0.17 (0.04–0.71) 0.015
Early initiation of breastfeeding No Yes	5 (10.20) 44 (89.80)	10 (11.63) 76 (88.37)	21 (15.67) 113 (84.33)	23 (24.21) 72 (75.79)
OR, 95%CI *p* value	1 ref.	0.86 (0.28–2.69) 0.800	0.61 (0.22–1.72) 0.352	0.36 (0.13–1.00) 0.051
ORa *, 95%CI *p* value	1 ref.	1.03 (0.27–3.91) 0.960	0.78 (0.22–2.80) 0.700	0.55 (0.15–2.06) 0.374
Small for gestational age No Yes	46 (90.20) 5 (9.80)	77 (88.51) 10 (11.49)	118 (88.06) 16 (11.94)	90 (90.91) 9 (9.09)
OR, 95%CI *p* value	1 ref.	1.19 (0.38–3.71) 0.758	1.25 (0.43–3.60) 0.683	0.92 (0.29–2.90) 0.887
ORa *, 95%CI *p* value	1 ref.	1.44 (0.40–5.19) 0.577	1.47 (0.41–5.20) 0.552	1.26 (0.32–5.00) 0.745
Low birth weight (<2500 g) No Yes	49 (96.08) 2 (3.92)	82 (93.18) 6 (6.82)	127 (94.78) 7 (5.22)	95 (95.00) 5 (5.00)
OR, 95%CI *p* value	1 ref.	1.79 (0.35–9.23) 0.485	1.35 (0.27–6.73) 0.714	1.29 (0.24–6.89) 0.766
ORa *, 95%CI *p* value	1 ref.	1.33 (0.23–7.64) 0.751	0.83 (0.13–5.18) 0.846	0.86 (0.12–6.06) 0.877
Preterm infant No Yes	50 (98.04) 1 (1.96)	86 (97.63) 2 (2.27)	130 (97.01) 4 (2.99)	95 (95.00) 5 (5.00)
OR, 95%CI *p* value	1 ref.	1.16 (0.10–13.15) 0.903	1.54 (0.17–14.10) 0.703	2.63 (0.30–23.14) 0.383
ORa *, 95%CI *p* value	1 ref.	1.21 (0.10–14.70) 0.880	1.56 (0.13–18.66) 0.725	2.63 (0.22–31.41) 0.444
Newborn health problems detected in the hospital No Yes	46 (90.20) 5 (9.80)	88 (100.00) 0 (0.00)	130 (97.01) 4 (2.99)	93 (93.00) 7 (7.00)
OR, 95%CI *p* value	1 ref.	1 (omitted)	0.28 (0.07–1.10) 0.068	0.69 (0.21–2.30) 0.549
ORa *, 95%CI *p* value	1 ref.	1 (omitted)	0.26 (0.05–1.24) 0.090	0.66 (0.13–3.31) 0.611
Newborn complications during the hospital stay No Yes	45 (88.24) 6 (11.76)	82 (93.18) 6 (6.82)	118 (88.06) 16 (11.94)	89 (89.00) 11 (11.00)
OR, 95%CI *p* value	1 ref.	0.55 (0.17–1.80) 0.322	1.02 (0.37–2.76) 0.974	0.93 (0.32–2.67) 0.888
ORa *, 95%CI *p* value	1 ref.	0.95 (0.25–3.69) 0.942	1.56 (0.44–5.49) 0.493	1.64 (0.42–6.38) 0.478
Newborn hospital admission No Yes	48 (94.12) 3 (5.88)	86 (97.73) 2 (2.27)	126 (94.03) 8 (5.97)	91 (91.00) 9 (9.00)
OR, 95%CI *p* value	1 ref.	0.37 (0.06–2.31) 0.288	1.02 (0.26–3.99) 0.982	1.58 (0.41–6.12) 0.506
ORa *, 95%CI *p* value	1 ref.	0.64 (0.08–4.92) 0.671	2.00 (0.36–11.11) 0.428	2.80 (0.48–16.42) 0.253
Exclusive breastfeeding in the first two months of life No Yes	34 (66.67) 17 (33.33)	47 (53.41) 41 (46.59)	56 (41.79) 78 (58.21)	59 (59.00) 41 (41.00)
OR, 95%CI *p* value	1 ref.	1.74 (0.85–3.57) 0.128	2.79 (1.42–5.48) 0.003	1.39 (0.69–2.81) 0.360
ORa *, 95%CI *p* value	1 ref.	1.36 (0.62–2.98) 0.435	1.94 (0.90–4.20) 0.091	1.08 (0.46–2.52) 0.863
Infant health problems during the first two months of life No Yes	47 (92.16) 4 (7.84)	87 (98.86) 1 (1.14)	128 (95.52) 6 (4.48)	88 (88.00) 12 (12.00)
OR, 95%CI *p* value	1 ref.	0.14 (0.01–1.24) 0.077	0.55 (0.15–2.04) 0.372	1.60 (0.49–5.24) 0.436
ORa *, 95%CI *p* value	1 ref.	0.13 (0.01–1.38) 0.091	0.43 (0.09–2.08) 0.297	1.15 (0.24–5.46) 0.857

* Adjusted for education level of the mother, income level, maternal smoking habit, previous history of miscarriage, type of delivery, and the presence of any pathology prior to pregnancy.

**Table 4:** Impact of maternal age on different continuous newborn variables.

Variable	Crude Analysis					Multivariate Analysis *				
	Age of Mother (Years) *m ± SEM*				*p* Value	Age of Mother (Years) *m ± SEM*				*p* Value
	<25	25–29	30–34	≥35		<25	25–29	30–34	≥35	
**Newborn weight (g)**	3277.45 ± 56.49	3303.07 ± 50.62	3244.85 ± 39.44	3215.85 ± 97.95	0.738	3287.12 ± 69.07	3310.56 ± 48.95	3256.70 ± 39.62	3223.41 ± 49.42	0.776
**Newborn head circumference (cm)**	34.56 ± 0.28	34.61 ± 0.30	34.43 ± 0.22	34.32 ± 0.22	0.910	34.25 ± 0.40	34.71 ± 0.29	34.52 ± 0.23	34.39 ± 0.29	0.348
**Newborn length (cm)**	49.13 ± 0.44	49.54 ± 0.35	49.5 ± 0.22	49.52 ± 0.25	0.405	49.30 ± 0.46	49.61 ± 0.32	49.48 ± 0.27	49.45 ± 0.33	0.585
**Apgar score at 1 min.**	8.94 ± 0.05	8.92 ± 0.04	8.79 ± 0.05	8.79 ± 0.06	0.825	8.88 ± 0.09	8.91 ± 0.06	8.81 ± 0.05	8.80 ± 0.06	0.746
**Apgar score at 5 min.**	9.53 ± 0.07	9.53 ± 0.05	9.40 ± 0.05	9.35 ± 0.05	0.960	9.53 ± 0.08	9.54 ± 0.06	9.40 ± 0.05	9.36 ± 0.06	0.993

* Adjusted for education level of the mother, income level, maternal smoking habit, previous history of miscarriage, type of delivery, and the presence of any pathology prior to pregnancy. Abbreviations: *m*, mean; SEM, standard error of mean.
